# Carbon-based composites for rechargeable zinc-air batteries: A mini review

**DOI:** 10.3389/fchem.2022.1074984

**Published:** 2022-11-16

**Authors:** Yuzhen Liu, Junjie Lu, Shaofeng Xu, Wei Zhang, De Gao

**Affiliations:** School of Mechatronics and Energy Engineering, Ningbo Tech University, Ningbo, China

**Keywords:** zinc-air batteries, ORR, OER, electrocatalysts, carbon-based composites

## Abstract

Rechargeable zinc-air batteries (ZABs) have gained a significant amount of attention as next-generation energy conversion and storage devices owing to their high energy density and environmental friendliness, as well as their safety and low cost. The performance of ZABs is dominated by oxygen electrocatalysis, which includes the oxygen reduction reaction (ORR) and the oxygen evolution reaction (OER). Therefore, it is crucial to develop effective bifunctional oxygen electrocatalysts that are both highly active and stable. Carbon-based materials are regarded as reliable candidates because of their superior electrical conductivity, low price, and high durability. In this Review, we briefly introduce the configuration of ZABs and the reaction mechanism of bifunctional ORR/OER catalysts. Then, the most recent developments in carbon-based bifunctional catalysts are summarized in terms of carbon-based metal composites, carbon-based metal oxide composites, and other carbon-based composites. In the final section, we go through the significant obstacles and potential future developments for carbon-based bifunctional oxygen catalysts for ZABs.

## Introduction

The increasing consumption of fossil fuels as a result of growing energy demand has exacerbated the development of environmental issues. Environmental problems are particularly evident in a developing country with coal as its primary energy source. Challenges from the energy and the environment sector are driving people to constantly seek new energy sources and innovative energy storage and transformation. One of the most practical and efficient energy conversion and storage methods is electrochemical energy conversion and storage technologies (such as metal-air cells, fuel cells, batteries, and supercapacitors). Rechargeable metal-air batteries have gained popularity in recent years owing to their low cost, environmental friendliness, and high safety. In virtue of high specific energy density, with a theoretical energy density of 1,350 kWh kg^−1^, zinc-air batteries (ZABs) have emerged as the most promising new energy battery for the next generation (Chen et al., 2020; Sun et al., 2021). Current research in rechargeable ZABs is focused on the development of effective bifunctional catalysts to address the slow kinetics of oxygen in the processes of oxygen reduction reaction (ORR) and oxygen evolution reaction (OER), to enhance the battery’s charge and discharge efficiency and decrease energy loss; efforts have also been made to find alternative materials to precious metal to reduce the cost of ZABs. However, the commercialization of secondary aqueous ZABs is still hindered: one of the core components of the battery—the air cathode that undertakes the OER and ORR reactions in the alkaline aqueous electrolyte, has oxidation/reduction reactions. The high reaction overpotential leads to the issues of low energy efficiency and unstable systems, and the electrode material must also meet several specifications, including overall electrical conductivity, oxygen diffusivity, volume space, and weight restrictions (Yin et al., 2021).

Due to their outstanding conductivity and diverse structural morphology, carbon-based materials have aided in the development of cathode materials for ZABs, such as light current collectors in gas diffusion layers (GDL) and conductive porous catalytic support materials (Wu et al., 2020b; Zhao et al., 2021). More notably, carbon-based materials can achieve synergistic effects through morphology composite design (Ying et al., 2022), defect engineering (Dong et al., 2020), and doping modification (Zhang et al., 2022a), acting as a catalytic layer (CL) and developing into a multifunctional cathode material. This results in enhanced bifunctional electrocatalytic OER/ORR reaction performance. Consequently, the benefits of carbon-based materials are mainly manifested in: 1) carbon-based materials come in a variety of structures, including 0D fullerenes, 1D carbon nanotubes (CNTs), 2D graphene, and 3D porous carbon nanostructure morphologies. These morphologies offer more exposed active areas and hasten mass transfer; 2) due to their excellent electrical conductivity, large specific surface area, superior corrosion resistance, and low cost, carbon-based materials have garnered a lot of scientific interest; and 3) fast charge transfer in redox processes can be ensured by the free electrons in the sp^2^ carbon structure. Furthermore, to improve catalytic activity, their electronic structures can be altered by heteroatom doping, defect engineering, or integration with other metals. These qualities have substantially enabled the traditional air cathode of rechargeable ZABs’ simplified design and established the ground for its ongoing commercialization. The study area exhibits a strong and increasing trend due to the growing utilization of carbon in ZAB cathode materials.

Currently, domestic and international research is primarily concerned with developing oxygen electrocatalysts in ZABs from the perspectives of carbon-based metal composites, carbon-based metal oxide composites, and other carbon-based composites. For example, suitable carbon-based catalysts are being sought after, as well as novel carbon-based nanomaterials that can be used to create high-efficiency bifunctional catalysts. The development of carbon-based cathode materials is influenced by various variables, such as optimization of design strategies, cost-effective synthesis techniques, and regulation of material properties. Each of these elements closely connects to the marketing of secondary ZABs. Therefore, it is imperative to analyze the most significant recent advancements to guide future research.

## Fundamentals of zinc-air batteries

**FIGURE 1:**
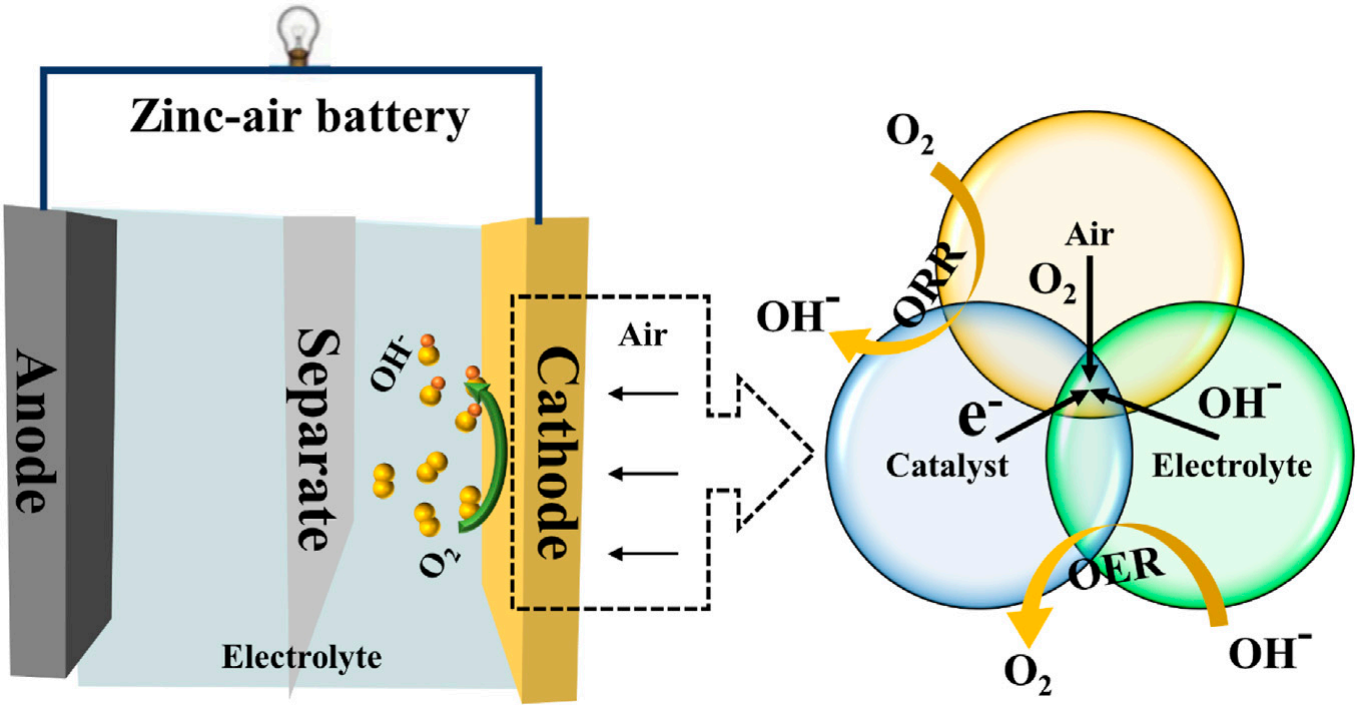
A schematic configuration of zinc-air batteries and the schematic diagram of the oxygen bi-functional electrocatalysts.

ZABs, as we are all aware, employ zinc metal as the negative fuel and oxygen from the air as the positive fuel. The amount of active material stored at both electrodes determines the battery’s energy output for conventional batteries. Since the positive part of a ZAB directly uses ambient oxygen as its active component, its energy is mostly controlled by a zinc plate that serves as the anode and is so frequently referred to as a “semi-fuel cell” (Liu et al., 2022b). The structural schematic diagram of a ZAB is depicted in Figure 1. The electrolyte, zinc plate functioning as the anode, and air electrode acting as the cathode are the main elements of ZABs. Zinc plate undergoes electrochemical oxidation on the anode, producing electrons as a result, which creates a current. The air electrode, which is the core of the ZABs, serves as the site for the catalytic ORR on the cathode. The oxygen from the external air first passes through the breathable layer of the air electrode. It then diffuses to the catalytic layer, where it undergoes a three-phase electrochemical reduction reaction takes place at the contact with the electrolyte. The high specific capacity of the ZABs can be obtained by using more zinc-negative electrodes since the catalytic electrode itself is not consumed during the process. As a result, the zinc anode for a ZAB is an energy storage device that determines the battery’s output capacity; for the positive portion, it effectively serves as an energy converter that determines the power output capacity of battery (Zhang et al., 2022b).

During discharge, Zn(OH)_4_^2−^ ions are generated when the reaction occurs between zinc and OH^−^ in the alkaline electrolyte. These zincate ions eventually oversaturate the electrolyte as the process continues, after which they break down into insoluble zinc oxide. The oxygen from the air is physically adsorbed onto the surface of the catalytic layer at the positive electrode of the ZABs by taking advantage of the large specific surface area of the catalytic layer. O_2_ then diffuses into the porous gas diffusion electrode, where the ORR takes place. As OH^−^ is produced, it moves to the zinc anode to finish the battery process (Chang et al., 2021). The electrochemical reaction process of the ZABs is shown as follows:

Anode: Zn − 2e^−^ = Zn^2+^
Cathode: O_2_ + 2 H_2_O + 4e^−^ = 4OH^−^
Overall: 1/2 O_2_ + Zn = ZnO

## Carbon-based composites

**FIGURE 2:**
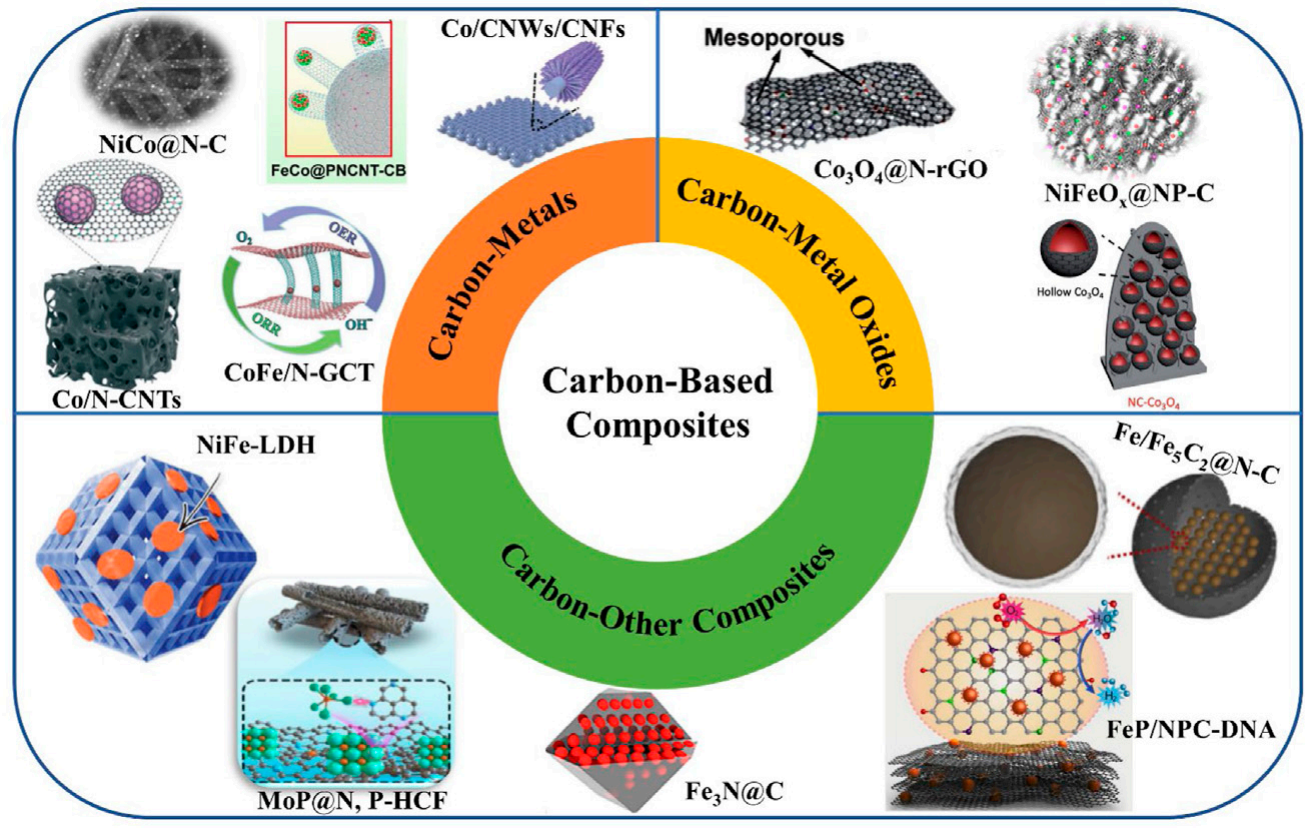
Summary of the carbon-based composites used to boost the electrochemical performances of zinc-air batteries. Reprinted with permission from (Guan et al., 2017; Wang et al., 2017; Fu et al., 2018; Li et al., 2018; Liu et al., 2018; Xia et al., 2021) with permission from WILEY-VCH. Reprinted with permission from (Chen et al., 2018; Song et al., 2019; He et al., 2021; Radwan et al., 2021; Yu et al., 2022; Zhu and Zhang, 2022) with permission from Elsevier. Reprinted with permission from (Chen et al., 2022) with permission from The Royal Society of Chemistry.

The mass transfer on the catalyst surface is the primary determinant of the catalytic performance, as the catalytic process occurs on the catalyst surface and interface. Additionally, OER and ORR typically involve electron transport and gas/liquid diffusion. Accordingly, the electrolyte/oxygen mass transfer and the electrical conduction between the active site and the electrode should be taken into account when designing the structural elements of ORR and OER electrocatalysts (Chang et al., 2021; Peng et al., 2022). As shown in Figure 2, the most recent advancements in carbon-based bifunctional catalysts are briefly covered in this Review from the perspective of carbon-based metal composites, carbon-based metal oxide composites, and other carbon-based composites.

### Carbon-metal composites

Based on catalysts consisting of noble metals, carbon-metal electrocatalysts have been created. Noble metals have a unique electronic configuration that can simply adsorb reactant molecules to create catalytic active centers, as their outermost orbitals are not filled with electrons or are not completely filled with electrons. In earlier investigations, traditional Pt/C catalysts have received much attention. For instance, through a polyol route, Sharma et al. (2021) created Pt/C electrocatalysts *via* a simple microwave pretreatment of the carbon support. The produced active materials outperformed commercial catalysts.

In metal-air battery catalysts, the replacement of platinum by transition metals with high reserves and a low degree of refinement has gradually attracted widespread attention. Among these, the use of Cu, Fe, Co, Ni, and their alloys in the field of ZABs has drawn increasing amounts of study attention and has been thoroughly investigated both experimentally and conceptually (Chen et al., 2021; Liu et al., 2021; Chen et al., 2022). For instance, Liu et al. (2016) evaluated the catalytic performance of transition metal nanoparticles (Fe, Co, and Ni) encapsulated in a nitrogen-doped carbon nanotube. The results showed that the Co nanoparticles in CNTs delivered the highest bifunctional catalytic activity for ORR and OER, good selectivity, and durability. The half-wave potential (0.86 V) of the optimized catalyst exceeded that of the state-of-the-art Pt/C, and the overpotential (0.39 V, 10 mA cm^−2^) of the catalyst was comparable to that of the RuO_2_ catalyst. Recently, Yang et al. (2022) designed ultrafine iron nanoparticles encapsulated in functional carbon nanotubes as an electrocatalyst. The ZABs demonstrated excellent open-circuit voltage (1.45 V), power density (106.5 mW cm^−2^), and steady cycling capability benefited from the ability of Fe nanoparticles to stimulate the adsorption and activation of reactive oxygen species on active sites, as well as to increase the O adsorption capacity. Electrospinning technology was also adopted to prepare the Co/CNWs/CNFs active catalysts, which exhibited excellent bifunctional and robust oxygen electrocatalytic performance due to the hierarchical structure with abundant active components (Xia et al., 2021). The assembled rechargeable ZABs yielded a high-power density of 304 mW cm^−2^ and excellent cycling stability for over 1,500 h at 5 mA cm^−2^.

In addition to single metal nanoparticles, metal alloying is a superior option as electrocatalysts for ZABs, such as CoFe (Wu et al., 2020a; Zhu and Zhang, 2022) and NiCo alloy (Fu et al., 2018). Fu et al. (2018) constructed a hybrid catalyst of NiCo alloy nanoparticles embedded in nitrogen-doped carbon nanofibers by electrospinning and post-calcination. The hybrid catalyst NiCo@N-C showed excellent catalytic performance for ORR and OER, even outperforming commercial Pt/C and RuO_2_ catalysts, respectively. For morphology design, 3D catalysts were developed, which are composed of CoFe alloy nanoparticles in N-doped CNTs entangled with rGO. This catalyst exhibited remarkable catalytic activity in ZABs with a high open-circuit voltage (1.43 V), a steady discharge voltage (1.22 V), a high energy efficiency (60.1%), and good stability (1,600 cycles at 10 mA cm^−2^) (Liu et al., 2018). In short, the enhanced catalytic activity of the composite catalyst, including decreased intrinsic resistance and charge transfer resistance, is primarily attributable to the synergistic interaction between the alloy nanoparticles and the carbon matrix.

By maximizing the use of atoms, creating more active sites, and learning more about the catalytic mechanism, carbon-metal composites with atomically distributed active sites have attracted increasing attention (Wagh et al., 2020; Luo et al., 2021; Wu et al., 2022). For instance, a catalyst made of Cu atomically dispersed in N-doped carbon displayed remarkable ORR and kinetic performance in an alkaline solution (Jin et al., 2021). To realize the atomic dispersion of Fe-N on N and S co-decorated hierarchical carbon layers and produce single-atom bifunctional catalysts, Chen et al. (2017) developed a new S-containing method. The abundance of atomically distributed Fe-N species, the hierarchical structure’s increased options for active sites, and the electrical conductivity are all factors that contribute to the material’s high catalytic activity. Most recently, the dual single-atomic Co/Ni sites catalysts were reported, where P and N atoms direct coordinated with atomically dispersed Co and Ni species (Hu et al., 2021).

Simply stated, the strong coupling between metal and carbon materials, which can efficiently change the local electronic structure and thereby optimize the intermediate adsorption, is responsible for the electrocatalytic activity of carbon-metal composites. Because of the intricate nature of various nanostructures and metal coordination compositions, however, there has not been any concrete proof of metal involvement in the system to date. With the help of theoretical simulations, sophisticated characterization methods, and controllable fabrication of targeted active sites with atomic precision, more work must still be done to definitively identify the nature of the active sites.

### Carbon-metal oxide composites

Metal oxides are of great interest due to their low cost, nontoxicity, and environmental friendliness. Since metal oxides may have various configurations and morphologies, their catalytic properties vary significantly. However, metal oxides have limitations, such as low electrical conductivity and small surface area. Therefore, the mixing of carbon materials with metal oxides can improve the electrical conductivity of metal oxide (MO)/C composites and form M-O-C bonds, which can facilitate the adsorption and desorption processes of intermediates in catalytic reactions (Wang et al., 2020).

Metal oxide-embedded carbon composites are multifunctional electrochemical catalysts. However, due to significant metal aggregation, the feasibility is compromised and its catalytic endurance is decreased. To alleviate the metal aggregation, a unique Al_2_O_3_ nanolayer confinement technique has been proposed to construct Co_3_O_4_ in a carbon matrix by directly pyrolyzing Zn/Co-ZIFs (Zhu et al., 2018). The protective Al_2_O_3_ layer offers significant advantages in capturing carbon and nitrogen molecules, reducing the agglomeration of Co species, and ultimately improving catalytic performance. Guan et al. (2017) reported a bifunctional electrocatalyst consisting of hollow Co_3_O_4_ nanospheres in nitrogen-doped carbon nano-wall arrays. The carbon onion coating suppressed the Kirkendall effect to facilitate the transformation of Co nanoparticles into irregular hollow oxide nanospheres with fine nanoparticle structures. Li et al. (2018) designed atomically thin mesoporous Co_3_O_4_ layers coupled with N-reduced graphene oxide nanosheets as catalysts for flexible fibrous ZABs. Compared with Pt/C + RuO_2_ ZABs, the Co_3_O_4_ layer-based ZABs exhibited better discharge and charge polarization properties and stable cycling stability. The inherent electronic and/or surface structures of metal oxides can be significantly changed further with metal/metal oxides to enhance electrochemical activity. In this regard, Xiao et al. (2020) fabricated excellent FeOx/Fe heterostructures embedded in N-doped carbon frameworks. Highly scattered N-doped carbon three-dimensional porous structures contain tunable FeOx/Fe heterostructures with many exposed crystal planes. Additionally, the performance of the ORR can be significantly enhanced by using the proper H_2_ treatment temperature. In addition to single metal oxide, spinel iron-cobalt oxide was reported that encapsulated in nitrogen-doped ordered mesoporous carbon for rechargeable metal-air batteries, exhibiting excellent activity with a half-wave potential of 0.89 V (Wei et al., 2021). Based on recent studies, the improved ORR/OER activity may have been caused by multiple factors, including enhanced electrical conductivity, altered electronic structure, and modified crystal structure, which were all attributed to carbon-metal oxide composites.

### Carbon-other composites

Other transition metal compounds, such as metal carbides (Liu et al., 2022a), hydroxides (Zhang et al., 2021; Ambriz-Pelaez et al., 2022), chalcogenides, nitrides (Radwan et al., 2021), and phosphides (Vijayakumar et al., 2022; Yu et al., 2022), have also been investigated as electrocatalysts in addition to metals and the alloy compounds, as well as metal oxides. Through logical control of neighboring anions, the electronic structure of metal cations can be altered to enhance the electrocatalytic activity.

For example, Fe_3_N nanoparticles encapsulated in a nitrogen-rich doped carbon framework were prepared *via* a hydrothermal method combined with pyrolysis under ammonia gas (Radwan et al., 2021). The designed electrocatalysts showed a higher half-wave potential (25 mV) than the carbon-supported platinum catalyst in an alkaline medium. As an air electrode for ZABs, it achieved a peak power density of 115.8 mW cm^−2^ and excellent durability compared to carbon-supported platinum. The synergistic interaction of chemical structures, impressive surface area, numerous active sites, and highly nitrogen-doped conductive carbon framework have been largely credited for their excellent catalytic performance. Regarding metal phosphides, iron phosphide (FeP) is an attractive alternative to platinum due to its abundance and cheapness. Encapsulation of FeP with doped carbon can improve the conductivity and durability of the metallic phase, especially the enhanced catalytic activity through bilateral electron coupling. For instance, a simple one-pot method was used to prepare FeP-embedded N, P co-doped carbon composites from natural DNA, which possessed large surface area, high degree of graphitization, and abundant high-quality dopants (Yu et al., 2022). Strikingly, long-chain DNA macromolecules constrain the growth of FeP to provide ultra-fine but highly crystalline nanoparticles. Therefore, the designed FeP possessed excellent alkaline ORR properties and (hydrogen evolution reaction) HER activity that was on par with Pt/C. To understand the effects of interfacial interactions on the ORR in ZABs, He et al. (2021) manufactured nitrogen and phosphorus co-doped molybdenum phosphide nanoparticles (MoP) hollow carbon fibers as novel bifunctional electrocatalysts. Strong electron transport is accomplished at the interface due to the high interfacial contacts between the carbon and MoP nanoparticles. This enables the homogeneous binding of nanoparticles to the doped carbon matrix, thereby promoting the oxygen electrode reaction kinetics.

## Summary and outlook

Carbon-based materials provide rich solutions for the rational design of air electrodes and can extend to other metal-air battery systems. Despite many academic achievements have been made, challenges still remain. The prerequisites for large-scale application of secondary zinc air batteries depend largely on the stability of the air electrode carrying the gas-liquid-solid three-phase reaction. For example, flexible ZABs, need to maintain the effective contact and flow of both oxygen and electrolyte on the catalytically active surface under deformation conditions, while avoiding the separation of catalytically active materials; in the slow charging process at high anode potential, the corrosion of carbon can lead not only to the degradation of the air cathode but also lead to the carbonation of the electrolyte. Therefore, the stability in practice needs to be further demonstrated; in addition, the microstructure of carbon materials needs to be studied in more detail to support more efficient three-phase reactions and the cost of producing materials should also be considered.

In the future, follow-up efforts can be made in three areas: 1) considering that a variety of circumstances can alter the catalytic performance, it is necessary to establish the evaluation criteria for material properties to guide the rational design of high-efficiency electrocatalysts through reasonable control strategies; 2) to comprehend the reaction mechanism, more work must be put into monitoring the intricate oxygen-catalyzed reactions. Advanced *in-situ* characterization techniques combined with theoretical modeling can be used to reveal the reactivity of oxygen catalysts at the atomic level and guide the rational design of high-performance electrocatalysts; and 3) in commercially viable ZAB devices, catalysts that exhibit great activity in the lab may not behave well, especially at high current densities and operating temperatures. It is recommended that catalysts are tested in real-world settings to create a comparable connection between laboratory results and practical applications.
